# Prediction of chronic kidney disease after acute kidney injury in ICU patients: study protocol for the PREDICT multicenter prospective observational study

**DOI:** 10.1186/s13613-018-0421-7

**Published:** 2018-07-06

**Authors:** Guillaume Geri, Bénédicte Stengel, Christian Jacquelinet, Philippe Aegerter, Ziad A. Massy, Antoine Vieillard-Baron, the PREDICT investigators, Stéphane Legriel, Virginie Laurent, Jean-Louis Teboul, Anatole Virginie Tarazona, Armand Mekontso-Dessap, Jean-Paul Mira, Jean-Luc Diehl, Romain Pirracchio, Naike Bigé, Claire Dupuis, Stéphane Gaudry, Julien Maizel, Bertrand Souweine, Lara Zafrani, Bruno Mégarbane, Alexandre Mebazaa, Antoine Durbach, Vincent Audard, Eric Thervet, Jean-Jacques Boffa, Guillaume Hanouna, Dimitri Titeca, Carole Philiponnet, Denis Glotz

**Affiliations:** 1 Medico-Surgical ICU, Service de Réanimation médico-chirurgicale, Ambroise Paré Hospital, APHP, 92100 Boulogne Billancourt, France; 2 Inserm U1018, Center for Research in Epidemiology and Population Health (CESP), Univ Paris Sud, Univ Paris Saclay, Villejuif, France; 3 Versailles Saint Quentin University, Montigny le Bretonneux, France; 4 Biomedicine Agency, Saint Denis, France; 5 Department of Clinical Research and Public Health, Ambroise Paré Hospital, APHP, Boulogne Billancourt, France; 6 UVSQ-INSERM U1168, University Paris Saclay, Villejuif, France; 7 Department of Nephrology, Ambroise Paré Hospital, APHP, Boulogne Billancourt, France

**Keywords:** Chronic kidney disease, Acute kidney injury, Intensive care unit, End-stage renal disease

## Abstract

**Background:**

Acute kidney injury (AKI) is frequent and associated with poor outcome in intensive care unit (ICU) patients. Besides the association with short- and long-term mortality, the increased risk of chronic kidney disease (CKD) has been recently highlighted in non-ICU patients. This study aims to describe the incidence and determinants of CKD after AKI and to develop a prediction score for CKD in ICU patients.

**Methods:**

Prospective multicenter (*n* = 17) observational study included 1200 ICU patients who suffered from AKI (defined by an AKIN stage ≥ 1) during their ICU stay and were discharged alive from ICU. Preexisting end-stage renal disease (ESRD) and immunosuppressant treatments are the main exclusion criteria. Patients will be monitored by a nephrologist at day 90 and every year for 3 years. The main outcome is the occurrence of CKD defined by a creatinine-based estimated glomerular filtration rate (eGFR) lower than 60 mL/min/1.73 m^2^ or renal replacement therapy for ESRD in patients whose eGFR will be normalized (≥ 60 mL/min/1.73 m^2^) at day 90. Secondary outcomes include albuminuria changes, eGFR decline slope and ESRD risk in patients with preexisting CKD, cardiovascular and thromboembolic events and health-related quality of life.

**Discussion:**

This is the first study prospectively investigating kidney function evolution in ICU patients who suffered from AKI. Albuminuria and eGFR monitoring will allow to identify ICU patients at risk of CKD who may benefit from close surveillance after recovering from AKI. Major patient and AKI-related determinants will be tested to develop a prediction score for CKD in this population.

*Trial registration* ClinicalTrials.gov, NCT03282409. Registered on September 14, 2017

## Background

 Acute kidney injury (AKI) occurs very frequently in patients admitted to an intensive care unit (ICU). The prevalence of AKI in ICU patients has been estimated between 5 and 15% [1–3]. It is strongly and independently associated with short- and long-term mortality [4]. While acute tubular necrosis is the most frequently reported cause of AKI in ICU patients, numerous pathological patterns have been observed in autopsy of kidneys in patients who died in the ICU following a septic shock [5]. This highlights the complexity of AKI in ICU, related to many harmful factors from ischemia–reperfusion lesions to sepsis-specific injuries. This may explain the association with AKI occurrence in the ICU and long-term mortality as some of these injuries are not as able to regenerate as tubular cells [3]. Moreover, endothelial dysfunction may be a worsening factor impacting long-term renal recovery and so the occurrence of chronic kidney disease.

Besides an association between AKI occurrence and long-term arterial hypertension, AKI has also been associated with chronic kidney disease (CKD), even if glomerular filtration rate (GFR) rose back to normal range [6, 7]. However, most of these data come from administrative registries and mostly include non-ICU patients. In the ICU setting, Wald et al. [8] reported an incidence of end-stage renal disease (ESRD) of 2.63 and 0.91 cases per 100 ICU person-year in patients with AKI requiring dialysis during the ICU stay, and in matched controls without AKI, respectively. While it is well recognized that an early management of CKD patients may slow GFR decline, the identification of AKI patients at risk to develop CKD is uneasy.

Thus, we aim to develop a prediction score for CKD in patients who suffered from AKI during their ICU stay and were discharged alive using a prospective multicenter observational study.

## Methods/design

### Design and setting

The prediction of chronic kidney disease after acute kidney injury in the intensive care unit (PREDICT) study is a multicenter observational prospective study promoted by the Assistance Publique—Hôpitaux de Paris. The study aims to evaluate factors associated with CKD risk in patients discharged alive from ICU and who suffered from AKI during ICU stay. The study was designed by both the medico-surgical intensive care unit and the department of clinical research and public health of Ambroise Paré Hospital (Boulogne-Billancourt, France) and the INSERM (French National Institute of Medical Research) Unit 1018 (Villejuif, France).

**Fig. 1:**
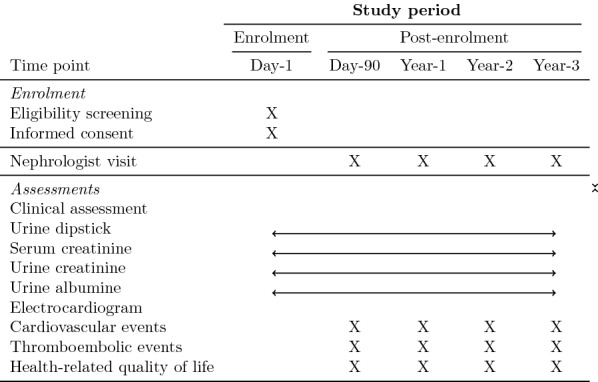
SPIRIT diagram

Patients who comply with the inclusion criteria will be monitored by a nephrologist at day 90 after ICU discharge and annually thereafter during 3 years: creatinine-based GFR estimation (eGFR), albuminuria measurement, cardiovascular events as well as health-related quality of life will be collected at each time point as shown in the Standard Protocol Items: Recommendations for Interventional Trials (SPIRIT) 2013 diagram (Fig. 1).

### Ethical considerations

This study follows the principles of the Helsinki Declaration 2008. The whole protocol has been reviewed and approved by the *Comité de Protection des Personnes*—*Sud*-*Est III* (no. 2017-053B).

### Registration

The study is registered on Clinical Trials (NCT03282409) and on the European Clinical Trials Database (*EudraCT 2017*-*A02649*-*44*).

### Participants

The study will enroll adults (18 years or older) discharged alive from ICU and who suffered from AKI during ICU stay. Participants will be eligible if they meet the acute kidney injury network (AKIN) classification stage 1 or higher [9]. All patients will have to be able to read and understand the patient information form and provide written informed consent. Patients will not be eligible for enrollment if (1) they suffer ESRD (defined by an eGFR below 15 mL/min/1.73 m^2^ or renal replacement therapy, i.e., dialysis or renal transplantation) before the index hospitalization, (2) they require renal replacement therapy at ICU discharge, (3) if they were prescribed immunosuppressive agents before index hospitalization or (4) there was no extra-renal failure at the time of acute kidney injury (defined by a SOFA score without the renal component equal to 0). Other exclusion criteria are expected life expectancy less than 90 days, inability to perform follow-up (homeless patients, main address far away from the nephrology center, etc.), pregnancy, privation of liberty by administrative or judicial decision, no affiliation to the national social security scheme and refusal to participate in the study.

### Recruitment, inclusion and consent

Patients will be recruited at ICU discharge. After a first screening with regard to the inclusion and exclusion criteria, patients who fulfill the inclusion criteria and are willing to participate will be included in the study after providing written informed consent. Patients will be recruited from 17 ICU (15 in the Greater Paris area, 1 in Clermont-Ferrand and 1 in Amiens) and monitored in 10 nephrology centers.

### Confidentiality

All original records will be kept on file at the trial sites or the coordinating data center for 15 years. The frozen trial database file will be kept on file for 15 years.

### Sample size

The sample size was calculated based on the primary outcome, namely the occurrence of de novo CKD at 3 years. Assuming an attrition bias of 10% and a competing risk of death of 20%, we calculated that 1200 patients fulfilling the inclusion criteria should be included according to the following distribution: 700 AKI patients who would fully recover kidney function at ICU discharge, 150 with impaired kidney function at ICU discharge and 350 with preexisting CKD. According to epidemiological data provided by the multicenter prospective observational study CUB-REA [10] and assuming an inclusion rate of 50% of the patients fulfilling the inclusion criteria, we estimated that the inclusion pace could be 5 patients/center/month leading to study completion within 24 months.

### Nephrologist active follow-up

Every patient will be monitored by a nephrologist at day 90 after ICU discharge and then every year for 3 years. As shown in the SPIRIT diagram, CKD will be evaluated by creatinine-based eGFR and urinary albumin (protein)-to-creatinine ratio. Cardiovascular and thromboembolic events will be collected as well as health-related quality of life.

### Primary and secondary outcomes

The main outcome of the study will be the incidence of CKD defined by an eGFR lower than 60 mL/min/1.73 m^2^ for at least 3 months or chronic dialysis initiation or renal transplantation, as previously published (KDIGO) in patients who recovered kidney function at day 90, defined by an eGFR ≥ 60 mL/min/1.73 m^2^.

The secondary outcomes will include:

- CKD progression defined by an eGFR decline greater than 30% at 3 years or the occurrence of ESRD (defined by an eGFR lower than 15 mL/min/1.73 m^2^ or chronic dialysis initiation or renal transplantation) in patients with preexisting CKD
- Evolution of urinary protein-to-creatinine ratio (mg/mmol)
- Prevalence of patients discharged from ICU with end-stage renal disease
- Occurrence of cardiovascular (acute coronary syndrome, ischemic stroke, peripheral artery disease, ventricular rhythm disorders, sudden death) and/or thromboembolic (deep-vein thrombosis or pulmonary embolism) events
- All-cause and cardiovascular long-term mortality
- Health-related quality of life using the KDQOL-SF-12 [11] and the EQ-5D-5L [12] questionnaires.

### Biological sampling

We plan to sample 10 mL of plasma at inclusion. This biobank will be stored at *the Centre de Ressources Biologiques* (Hôpital Ambroise Paré, APHP) and will be used to assess the association between biomarkers of kidney injury and long-term outcomes.

### Pre-planned ancillary analysis: 10-year follow-up

We plan to extend patient follow-up up to 10 years through record linkage between the PREDICT database and the following datasets:

- The national Renal Epidemiology and Information Network (REIN) registry to identify ESRD events (dialysis and kidney transplantation) [13]
- The national death registry (RNIPP, Registre national d’identité des personnes physiques) to assess vital status and identify cause(s) of death.
- The national health insurance information system (SNIIR-AM) to collect data about patient use of health resources.

Patient consent for this ancillary study will be requested at inclusion in the main study.

### Data collection, monitoring and data analysis

During the visits, data will be collected in an electronic case report form (eCRF) using CleanWEB software (TéléMédecine^®^, Boulogne, France). This data management system allows direct data entry. Data entry will be monitored by an independent researcher according to a predefined monitoring plan. Patient confidentiality will be ensured by using identification numbers.

Patient baseline characteristics will be compared between AKIN categories at inclusion using analysis of variance or Mann–Whitney test and Pearson’s Chi-square test for continuous and categorical data, respectively. Factors associated with main outcome will be evaluated using a competing risk survival analysis. Factors associated with the main outcome in univariate analysis with a *p* value lower than 0.10 will be included in the multivariate model. Clinically relevant interactions will be studied (especially with age, gender and preexisting diabetes). Factors associated with long-term mortality will be evaluated using the same methodology.

Yearly eGFR assessment will allow to describe the evolution of GFR over time and the evaluation of factors associated with eGFR decline. This analysis will be performed using mixed models with random intercepts and slopes.

The score aiming at predicting the occurrence of CKD at 3 years will be built as follows: (1) evaluation of factors associated with the main outcome using competing risk analysis, (2) assessment of the selection of the variables using the least absolute shrinkage and selection operator (LASSO) method, (3) evaluation of score performance, (4) assessment of the internal validity using bootstrapping. Such a score will be developed using two-thirds of the entire cohort and validated (external validation) using the remaining one-third.

The findings of this study will be published in national and international journals.

## Discussion

The PREDICT study is a multicenter observational prospective study aiming at describing the occurrence of CKD at 3 years in patients who suffered AKI during their ICU stay. It will be the first prospective study including a large sample of patients discharged alive from ICU able to provide CKD incidence after AKI in ICU and to highlight factors associated with CKD occurrence in this specific setting.

Several observational studies performed in medicine wards using administrative billing codes have reported an increased risk of CKD occurrence after AKI. While pathogenesis of such a mid- and long-term complication remains unclear, several hypotheses have been raised to explain such an outcome. Ischemia–reperfusion phenomena as well as tubular injuries, renal arteriolar injuries and endothelial dysfunction might explain the occurrence of long term of CKD after AKI [5]. All these phenomena widely occur in ICU patients, much more often than in non-ICU patients, and this could lead to a higher risk of CKD in this very specific subgroup of patients. Although clinically relevant, data about CKD after AKI in ICU patients remain scarce and specifically focused on ESRD occurrence and renal replacement therapy [14–16]. The present study may provide interesting insights in the early identification of these patients who could benefit from long-term prophylactic measures.

We will specifically focus on patients who recovered a normal eGFR. Kidney function recovery is difficult to define as eGFR may only be a part of such an evaluation. “De novo” CKD after AKI highlights the lack of discrimination of eGFR in CKD prediction in these patients and the need for new tools to identify patients who could benefit from prophylactic treatments [17]. Accordingly, the present study will provide two major information: (1) the proportion of patients who recover a normal eGFR and who will suffer CKD after ICU discharge and the factors (during and after the ICU stay) associated with this complication and (2) the kinetics of eGFR decline and more importantly of albuminuria, which may be an earlier biomarker of kidney injury. Thus, we aim to provide tools to help physicians to identify patients who will suffer CKD and who could be difficult to early target with the blood creatinine assessment. Furthermore, we could get some interesting results in the clinical, biological and pathological description of AKI injuries in ICU patients. Besides tubular injuries, numerous other lesions could be observed and could help to better understand chronic kidney injury pathogenesis.

Finally, we will also include patients with previously known CKD and will be able to describe eGFR decline slope after AKI in ICU. This could help nephrologists and transplantation specialists to model the kidney function worsening and prepare patients to dialysis or renal transplantation.

In conclusion, the PREDICT study will evaluate CKD incidence and its determinants in ICU patients who suffered AKI during their ICU stay. It will provide a score able to predict CKD and help to identify patients who could benefit from early management and potential prophylactic treatments.

### Trial status

Patient recruitment has started on April 26, 2018.

## Abbreviations

AKI: acute kidney injury
ICU: intensive care unit
CKD: chronic kidney disease
ESRD: end-stage renal disease
GFR: glomerular filtration rate
